# Pre-existing anxiety and mood disorders and multisystem outcomes in young adults with chronic kidney disease

**DOI:** 10.1093/ckj/sfag174

**Published:** 2026-05-28

**Authors:** Lino Merlino, Francesco Rainone, James Tollit, Michael J Kalra, Sarah Williford, Francesca Rusconi, Graziana G Battini, Ross A Dunne, Philip A Kalra, Constantina Chrysochou

**Affiliations:** Manchester University, Department of Cardiovascular Sciences, Manchester, UK; Donal O’Donoghue Renal Research Centre, Salford Royal Hospital, Salford, UK; Vimercate Hospital, Department of Nephrology, ASST Brianza, Vimercate, Italy; Manchester University, Department of Cardiovascular Sciences, Manchester, UK; Donal O’Donoghue Renal Research Centre, Salford Royal Hospital, Salford, UK; Manchester University, Department of Cardiovascular Sciences, Manchester, UK; Donal O’Donoghue Renal Research Centre, Salford Royal Hospital, Salford, UK; Donal O’Donoghue Renal Research Centre, Salford Royal Hospital, Salford, UK; TriNetX Europe NV, Sint-Martens-Latem, Belgium; TriNetX Europe NV, Sint-Martens-Latem, Belgium; Vimercate Hospital, Department of Nephrology, ASST Brianza, Vimercate, Italy; Greater Manchester Dementia Research Centre, Greater Manchester Mental Health Foundation Trust, Manchester, UK; Geoffrey Jefferson Brain Research Centre, University of Manchester, UK; Manchester University, Department of Cardiovascular Sciences, Manchester, UK; Donal O’Donoghue Renal Research Centre, Salford Royal Hospital, Salford, UK; Manchester University, Department of Cardiovascular Sciences, Manchester, UK; Donal O’Donoghue Renal Research Centre, Salford Royal Hospital, Salford, UK

**Keywords:** anxiety, CKD, mood, morbidity, young adults

## Abstract

**Background:**

Anxiety and mood disorders (AMD) are common in young adults and may amplify medical risk in chronic kidney disease (CKD). However, their prognostic impact in young adults with moderate–severe CKD remains poorly defined. We investigated whether pre-existing AMD identify a subgroup of young CKD patients at increased risk of adverse clinical outcomes.

**Methods:**

Using the TriNetX Global Collaborative Network, we identified young adults aged 18–30 years with stage 3–4 CKD documented in the Electronic Health Records. Patients with documented AMD prior to CKD identification were compared with those without AMD. Cohorts were balanced using 1:1 propensity score matching. Time-to-event outcomes were analyzed using Kaplan–Meier methods and Cox proportional hazards models. Prespecified sensitivity analyses included restriction to patients with documented body mass index and a landmark analysis with delayed outcome assessment to address surveillance bias and follow-up heterogeneity.

**Results:**

Among 3876 eligible patients, 1356 had pre-existing AMD. After matching, 734 patients remained in each cohort. Over follow-up, AMD was associated with higher risks of cardiovascular outcomes (ischemic heart disease, arrhythmias, stroke, hypotension), renal outcomes (acute kidney injury, progression to dialysis), pneumonia, neurocognitive complications (acute confusional state, encephalopathy), and sleep disorders. In the primary matched analysis, all-cause mortality did not differ between groups [hazard ratio (HR) 1.00, 95% confidence interval (CI) 0.72–1.39]. In prespecified sensitivity analyses restricted to patients with sustained follow-up and delayed outcome assessment, AMD was associated with higher mortality risk (HR 1.92, 95% CI 1.21–3.03).

**Conclusions:**

In young adults with stage 3–4 CKD, pre-existing AMD identify a phenotype associated with increased multisystem morbidity. Although no difference in mortality was observed in the primary analysis, sensitivity analyses suggest a potential association with excess mortality among patients with sustained follow-up. These findings support integrating mental health assessment into CKD risk stratification and highlight the need for prospective studies on the long-term survival.

## Graphical Abstract

**Graphical Abstract:**
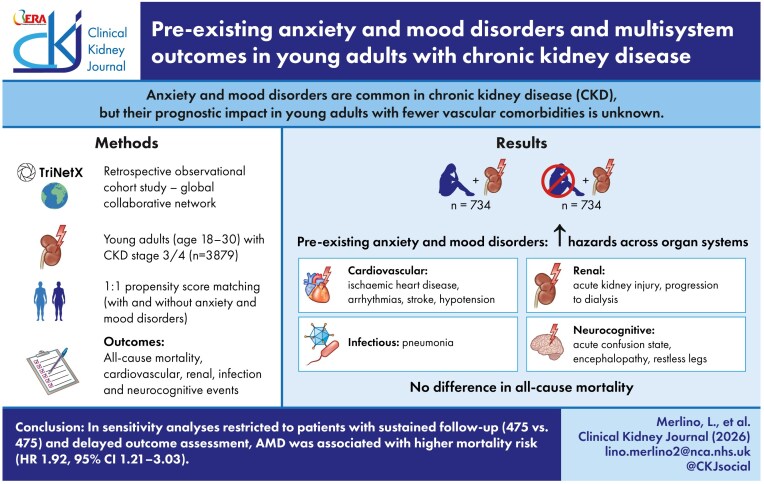

KEY LEARNING POINTS **What was known:**

- Anxiety and mood disorders (AMD) are common in chronic kidney disease (CKD), but prior studies focused on older adults with vascular comorbidity.
- Whether AMD contributes to morbidity in young CKD adults with fewer vascular confounders was unknown.
- Clarifying this link could identify AMD as a modifiable risk enhancer.

**This study adds:**

- Among young adults with stage 3–4 CKD, pre-existing AMD identifies a phenotype associated with increased cardiovascular, renal, infectious, and neurocognitive morbidity.
- Young adults with CKD, including those presenting directly to adult services, represent a high-risk group that may benefit from structured transitional and integrated care pathways, including mental health assessment.
- In patients with sustained follow-up, AMD was associated with higher mortality, suggesting a long-term risk signal.

**Potential impact:**

- Early identification of AMD could enable preventive, mental-health–informed CKD care.
- Integrating psychological screening into nephrology care may improve long-term outcomes and quality of life.
- Further prospective studies are needed to clarify whether AMD influences long-term survival in CKD.

## INTRODUCTION

Chronic kidney disease (CKD) is an increasing global health concern [1], linked to high rates of cardiovascular complications, neuropsychiatric disorders, and early death [2, 3]. Young adults with CKD are a particularly at-risk group, facing lifelong risks of adverse outcomes [4]. Identifying modifiable risk factors that affect their long-term prognosis is crucial.

According to the World Health Organization, 1:8 people in the world live with a mental disorder [5]. Mental disorders involve disturbances in thinking, emotional regulation, and behavior. Affective disorders are common mental health conditions that primarily include mood and anxiety.

Mood disorders, defined by abnormal mood changes from depression to mania, are common. The lifetime prevalence is ∼4%–19% for major depression, and 2.4%–2.8% for bipolar disorder. Women generally experience higher rates of most mood disorders, while bipolar disorder shows similar rates across sexes. These conditions tend to be heritable, often co-occur with anxiety and substance misuse and are a leading cause of disability worldwide, impacting social and occupational functioning [6].

Anxiety disorders, characterized by excessive fear or apprehension, are the most common mental health conditions, affecting 4% of the global population, which equals about 301 million people [7].

Anxiety and mood disorders (AMD) are common among patients with CKD, with estimates indicating rates two to three times higher than in the general population [8]. AMD are chronic, often recurrent, psychiatric conditions with substantial diagnostic overlap and frequent co-occurrence, reflecting a shared internalizing psychopathology [9]. Given that, we intentionally adopted a composite definition of AMD to reflect real-world psychiatric burden rather than isolated diagnostic categories.

We hypothesized that pre-existing AMD act as a cross-system risk amplifier in young adults with CKD, predisposing to adverse outcomes across cardiovascular, renal, infectious, and neurocognitive domains through shared biological, behavioral, and healthcare-interaction mechanisms [10–14].

Most studies have concentrated on older adults, advanced kidney disease, or dialysis patients [4], with mixed comorbidities, making it challenging to identify the specific role of AMD. Young adults with CKD, who are less likely to have extensive vascular comorbidities, form a group in which the relationship between AMD and CKD outcomes can be more clearly explored, avoiding confounders due to aging and burden of long-lasting medical conditions.

Understanding whether AMD confer additional risk for adverse outcomes in CKD patients may provide insights into mechanisms linking psychological and biological health. If AMD are associated with worse long-term outcomes in CKD, this would emphasize the importance of early detection and integrated care models in nephrology, especially considering that these patients are more likely to receive a transplant in the future.

We conducted a large-scale, propensity-matched cohort study using the TriNetX Global Collaborative Network (GCN) to compare outcomes in young adults with CKD stages 3–4, with and without pre-existing AMD.

## MATERIALS AND METHODS

### Study design and setting

We conducted a retrospective observational cohort study on CKD patients (stages 3–4) according to the KDIGO classification [15]. Data for this study were accessed through the TriNetX platform’s GCN [16]. TriNetX is a global research network aggregating anonymized electronic medical records from over 150 healthcare organizations, including academic centers, specialty and community hospitals and physicians’ groups. The network captures data from diverse insured and uninsured populations across geographic and socioeconomic settings and is continuously updated directly from participating EMR systems [17]. All data handling complied with applicable data protection regulations at contributing healthcare organizations, including General Data Protection Regulation GDPR (EU Regulation 2016/679) and HIPAA. Diagnoses were coded using ICD-10-CM, procedures using ICD-10-PCS or CPT, and laboratory data using Logical Observation Identifiers Names and Codes. As all data were fully anonymized, informed consent was waived.

### Patient populations

We used de-identified EMRs from the TriNetX GCN, comprising 147 HCOs worldwide. Queries were executed on 27 June 2025. We identified young adults aged 18–30 years with CKD stages 3–4 documented in the Electronic Health Records (EHR).

CKD stages 3–4 were defined by either a qualifying Estimated Glomerular Filtration Rate eGFR measurement between 15.0 and 59.9 ml/min/1.73 m² (CKD-EPI) followed by a confirmatory CKD event at least 3 months later, or a qualifying CKD diagnosis code (ICD-10-CM N18.3 or N18.4). As is typical in EHR-based studies, the earliest recorded evidence of CKD reflects the time of documentation rather than true disease onset; therefore, patients may have had variable durations of underlying CKD prior to cohort entry.

Prior documentation of eGFR >60 ml/min/1.73 m² was not required, as historical laboratory data availability varies across healthcare organizations.

Exclusions: to capture non–end-stage CKD at baseline, we excluded patients with evidence of kidney (or other solid-organ) transplantation or dialysis prior to the index event, using ICD-10-PCS, CPT, SNOMED, and related codes (e.g. renal transplantation procedures; Z94 transplant status; dialysis procedures/encounters and device complications).

### Cohort definitions

- Cohort 1 (AMD before CKD): patients with AMD recorded at any time prior to or on the CKD index event (ICD-10-CM F30–F39 and F40–F48) as per the Diagnostic and Statistical Manual of Mental Disorders [9], with the exposure window extending from the earliest available EHR entry within the TriNetX to CKD incidence.
- Cohort 2 (No AMD before CKD): patients with no recorded mood or anxiety/stress-related disorders up to and including the index window.

### Prespecified outcomes

Outcomes were selected *a priori* to represent clinically meaningful, acute, and reliably captured manifestations of multi-organ vulnerability rather than isolated disease entities. We did not assume uniform effects across outcomes but expected different levels of risk across organ systems sensitive to hemodynamic and inflammatory stress.

Mortality: All-cause mortality, identified by death status or ICD-10-CM R99.
Cardiovascular Outcomes: defined broadly to reflect the heterogeneous and often non-atherosclerotic cardiovascular manifestations in young adults with CKD.
Ischemic heart disease: ICD-10-CM I20–I25, encompassing ischemic syndromes beyond obstructive coronary disease.
Arrhythmias: Rhythm-related diagnoses including atrial fibrillation/flutter (I48), ventricular tachycardia (I47.2), and abnormalities of heart rhythm (R00), capturing clinically relevant rhythm disturbances requiring medical evaluation.
Stroke and cerebrovascular disease: I60–I69.
Transient ischemic attack: G45.
Hypotension: I95.
Renal outcomes:
  - Acute kidney injury (AKI): N17.
  - Progression to dialysis: initiation of hemodialysis or peritoneal dialysis (ICD-10-PCS, CPT, HCPCS, SNOMED).
Infectious outcome:
  - Pneumonia: J18, selected as a sentinel infection due to clinical relevance, biological plausibility, and reliable EMR capture. Outcomes with long latency or heterogeneous ascertainment (e.g. malignancy) were not included.
Neurocognitive and neurological outcomes:
  - Acute confusional states: amnestic disorder (F04), delirium (F05), and other/unspecified mental disorders due to physiological conditions (F06, F09), capturing clinically significant acute cognitive decompensation.
  - Encephalopathy: G93.40–G93.41.
  - Epilepsy/recurrent seizures: G40.
  - Sleep disorders: G47.
  - Restless legs syndrome: G25.81.

### Covariates and propensity score matching

To address confounding, propensity score matching (PSM) was performed using logistic regression with AMD (ICD-10 F30–F48) as the dependent variable. One-to-one nearest-neighbor matching without replacement was applied using a caliper of 0.1 SD. Covariates included demographics, kidney disease severity (eGFR), body mass index, cardiometabolic and respiratory comorbidities, health behaviors, psychiatric comorbidity, injury proxies, and healthcare utilization/socioeconomic factors (ICD-10 Z-codes). Covariate balance was assessed using standardized differences, with values <0.1 indicating adequate balance [18]. PSM was performed using available covariate data only; no imputation was applied, and patients with missing values for selected matching variables were excluded from matching. Psychotropic medications were not included as baseline covariates to avoid exposure misclassification and overadjustment for potential mediators on the causal pathway between AMD and downstream outcomes.

### Follow up and censoring

Outcomes were analyzed using time-to-event methods over a maximum follow-up of 10 years. Patients were followed from the CKD index date until the occurrence of the outcome of interest, the last recorded clinical encounter, or the end of data availability, with right censoring applied at the final documented encounter within the TriNetX network.

### Statistical analysis

Baseline characteristics are summarized as means (SD) for continuous variables, and counts (percentages) for categorical variables. Between-group comparisons used Student’s t-tests and Fisher’s exact tests. Time-to-event outcomes were analyzed using Kaplan–Meier methods and Cox proportional hazards regression, with right censoring at the last recorded clinical encounter. Hazard ratios (HRs) and 95% confidence intervals (CIs) were estimated from Cox models, with continuous covariates modeled linearly and categorical variables entered as indicator terms. Proportional hazards assumptions were assessed and not violated. Cumulative incidence and survival probabilities were estimated using Kaplan–Meier analysis. Patients with documented outcomes before the specified risk window were excluded. Prespecified sensitivity analyses involved restricting to patients with at least two healthcare encounters (visits) after the initial CKD event, separated by at least 30 days, within the observation period (from 1 day to 10 years post-index). Results were compared with the primary analysis for consistency. All tests were two-sided, with *P* < .05 considered statistically significant.

### Details of the software used to run the analyses

The TriNetX platform is a customized solution designed for use in the clinical research field. The statistical methods and software packages used to generate statistical analyses include the following: Java 11.0.16 (featuring Apache Commons Math 3.6.1), R 4.0.2 (with Hmisc1-1 and Survival 3.2-3), and Python 3.7 (incorporating lifelines 0.22.4, matplotlib 3.5.1, numpy 1.21.5, pandas 1.3.5, scipy 1.7.3, and statsmodels 0.13.2).

## RESULTS

**Figure 1:**
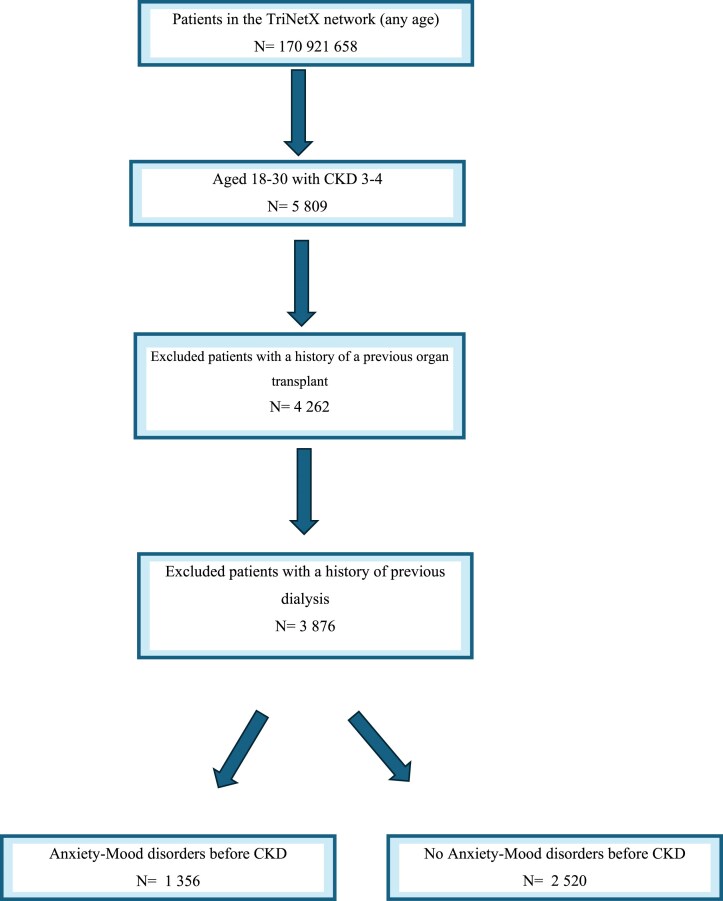
Patient cohorts’ definition.

Study population: Among 3876 young adults (18–30 years) with incident stage 3–4 CKD, 1356 had documented anxiety and/or mood disorders (AMD) prior to CKD onset and 2520 did not (Fig. 1). After 1:1 PSM, 734 patients remained in each cohort with excellent covariate balance (standardized mean differences <0.1; Tables 1 and 2).

**Table 1:** Baseline demographics of the two cohorts before and after PSM.

	Before PSM			After PSM		
Variable	AMD cohort (*n* = 1356)	No-AMD cohort (*n* = 2520)	St diff	AMD cohort (*n* = 734)	No-AMD cohort (*n* = 734)	St diff
Age at index, years (mean ± SD)	24.8 ± 3.8	24.7 ± 4.1	0.00	24.6 ± 3.8	24.8 ± 4.1	.04
eGFR (CKD-EPI), ml/min/1.73 m² (mean ± SD)	39.3 ± 19.0	38.6 ± 17.3	0.04	38.6 ± 18.2	39.4 ± 18.9	.04
BMI, kg/m² (mean ± SD)	28.7 ± 8.8 (n = 951)	27.8 ± 8.4 (n = 1,667)	0.10	27.6 ± 7.9 (n = 513)	28.1 ± 8.4 (n = 501)	.06
Female, *n* (%)	810 (59.7%)	1,138 (45.2%)	0.29	372 (50.7%)	367 (50.0%)	.01
Male, *n* (%)	546 (40.3%)	1,382 (54.8%)	0.29	362 (49.3%)	367 (50.0%)	.01
White, *n* (%)	832 (61.4%)	1,363 (54.1%)	0.15	423 (57.6%)	440 (59.9%)	.05
Black or African American, *n* (%)	352 (26.0%)	655 (26.0%)	0.001	207 (28.2%)	200 (27.2%)	.02
Asian, *n* (%)	50 (3.7%)	209 (8.3%)	0.19	32 (4.4%)	30 (4.1%)	.01
American Indian or Alaska Native, *n* (%)	16 (1.2%)	17 (0.7%)	0.05	10 (1.4%)	10 (1.4%)	<.001
Native Hawaiian or Other Pacific Islander, *n* (%)	14 (1.0%)	45 (1.8%)	0.06	11 (1.5%)	11 (1.5%)	<.001
Other race, *n* (%)	77 (5.7%)	186 (7.4%)	0.07	46 (6.3%)	38 (5.2%)	.05
Unknown race, *n* (%)	15 (1.1%)	45 (1.8%)	0.06	10 (1.4%)	11 (1.5%)	.01
Hispanic or Latino, *n* (%)	182 (13.4%)	326 (12.9%)	0.01	101 (13.8%)	92 (12.5%)	.04
Not Hispanic or Latino, *n* (%)	948 (69.9%)	1,734 (68.8%)	0.02	504 (68.7%)	522 (71.1%)	.05
Unknown ethnicity, *n* (%)	226 (16.7%)	460 (18.3%)	0.04	129 (17.6%)	120 (16.3%)	.03
BMI <18.5 kg/m², *n* (%)	160 (11.8%)	250 (9.9%)	0.06	89 (12.1%)	83 (11.3%)	.02
BMI 18.5–24.9 kg/m², *n* (%)	496 (36.6%)	864 (34.3%)	0.05	286 (39.0%)	253 (34.5%)	.09
BMI 25.0–29.9 kg/m², *n* (%)	380 (28.0%)	633 (25.1%)	0.07	207 (28.2%)	202 (27.5%)	.01
BMI ≥30 kg/m², *n* (%)	436 (32.2%)	635 (25.2%)	0.15	204 (27.8%)	206 (28.1%)	.01

St diff: standard difference. In pink background St diff >0.1.

**Table 2:** Baseline comorbidities of the two cohorts before and after PSM.

	Before PSM			After PSM		
Variable	AMD cohort (*n* = 1356)	No-AMD cohort (*n* = 2520)	St diff	AMD cohort (*n* = 734)	No-AMD cohort (*n* = 734)	St diff
Hypertension (I10), *n* (%)	778 (57.4%)	1,164 (46.2%)	0.22	377 (51.4%)	390 (53.1%)	0.03
Type 2 diabetes (E11), *n* (%)	269 (19.8%)	297 (11.8%)	0.22	118 (16.1%)	121 (16.5%)	0.01
Type 1 diabetes (E10), *n* (%)	210 (15.5%)	231 (9.2%)	0.19	98 (13.4%)	102 (13.9%)	0.07
Nicotine dependence (F17), *n* (%)	215 (15.9%)	149 (5.9%)	0.32	134 (18.3%)	129 (17.6%)	0.02
Asthma (J45), *n* (%)	201 (14.8%)	187 (7.4%)	0.24	89 (12.1%)	91 (12.4%)	0.01
Substance use disorders (F10–F19), *n* (%)	305 (22.5%)	215 (8.5%)	0.39	190 (25.9%)	180 (24.5%)	0.03
Other mental/behavioral disorders (F01–F99), *n* (%)	977 (72.1%)	388 (15.4%)	1.39	355 (48.4%)	352 (48.0%)	0.01
Injury/poisoning (S00–T88), *n* (%)	532 (39.2%)	603 (23.9%)	0.33	243 (33.1%)	233 (31.7%)	0.03
External causes of morbidity (V00–Y99), *n* (%)	234 (17.3%)	244 (9.7%)	0.22	110 (15.0%)	109 (14.9%)	0.00
Factors influencing health status (Z00–Z99), *n* (%)	1,041 (76.8%)	1,561 (61.9%)	0.33	535 (72.9%)	537 (73.2%)	0.01
Socioeconomic/psychosocial hazards (Z55–Z65), *n* (%)	89 (6.6%)	48 (1.9%)	0.23	31 (4.2%)	26 (3.5%)	0.03

St diff, standard difference. In pink background St diff >0.1.

Follow-up: Mean follow-up was 1827 (SD 1207) days in the AMD cohort and 1576 (SD 1199) days in the no-AMD cohort; median follow-up was 1741 and 1319 days, respectively, and was shorter than the maximum analytic window.

**Figure 2:**
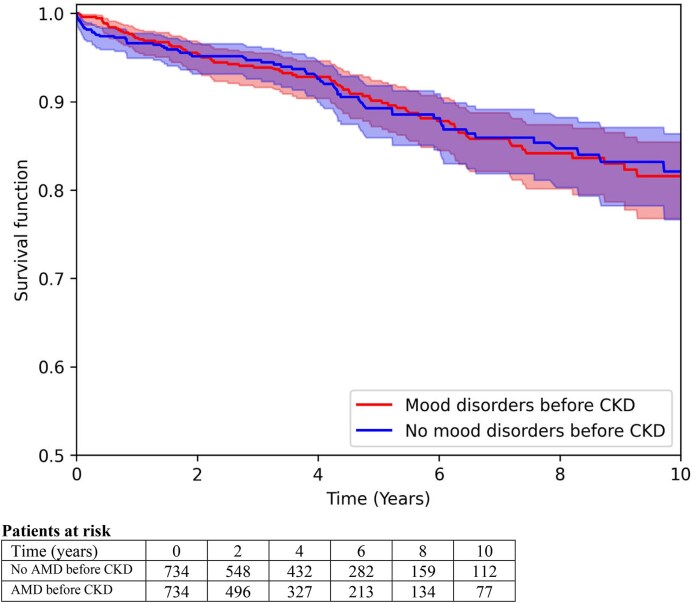
Survival function in the two groups over the space of 10 years.

**Figure 3:**
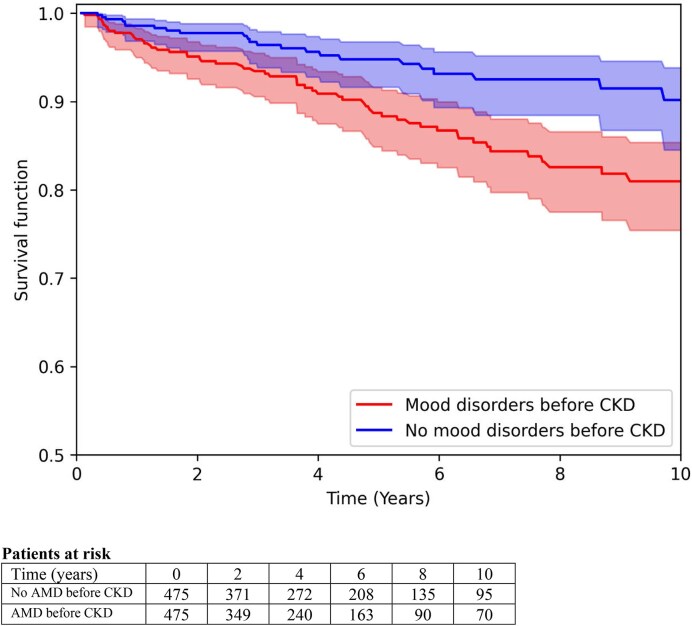
Survival function in the two groups over the space of 10 years (sensitivity analysis).

Mortality: In the primary matched analysis, all-cause mortality did not differ between groups (10.4% vs. 8.9%; HR 1.00, CI 0.72–1.39; *P* = .998) (Fig. 2). In the prespecified time-anchored subset analysis, patients with pre-existing AMD had a statistically significant higher hazard of death than matched controls, HR 1.92, CI 1.21–3.03, *P* = .004. (Figure 3 and Table 3).

**Table 3:** All-cause mortality in the prespecified landmark subset analysis (sensitivity analysis with sustained follow-up).

Outcome	AMD cohort	No-AMD cohort	Hazard ratio (95% CI), *P* value
All-cause mortality	58/475 (12.2%)	27/475 (5.7%)	1.92 (1.21–3.03), *P* = .004

Values are shown as number (%) of patients with at least one recorded event during follow-up. Hazard ratios are derived from Cox proportional hazards models with right-censoring. Outcome assessment began 30 days after CKD onset and was restricted to patients with documented longitudinal follow-up.

Cardiovascular outcomes: Pre-existing AMD was associated with higher hazards of ischemic heart disease (HR 1.75, CI 1.31–2.33), arrhythmia-related diagnoses (HR 1.69, CI 1.41–2.02), stroke (HR 1.66, CI 1.16–2.37), and hypotension (HR 1.76, CI 1.36–2.27). Transient ischemic attack did not differ between groups.

Renal outcomes: AMD was associated with higher hazards of AKI (HR 1.42, CI 1.23–1.64) and progression to dialysis (HR 1.59, CI 1.28–1.98).

Infectious outcomes: AMD was associated with an increased risk of pneumonia (HR 1.57, CI 1.19–2.07).

Neurological and sleep-related outcomes: AMD was associated with higher hazards of acute confusional states (HR 4.19, CI 2.18–8.04), encephalopathy (HR 2.06, CI 1.41–3.01), sleep disorders (HR 2.26, CI 1.77–2.88), and restless legs syndrome (HR 2.41, CI 1.07–5.42). Epilepsy did not differ significantly.

Sensitivity analyses: In the subset analysis (outcomes assessed from 30 days after CKD onset up to 3650 days), median follow-up was 1866 days in the AMD cohort and 1477 days in the no-AMD cohort. Associations, including mortality, were consistent with or stronger than those in the primary analysis.

Results are summarized in Table 4.

**Table 4:** Time-to-event analysis of prespecified secondary outcomes in the primary matched cohort (734) and subset analysis (475).

Outcome	AMD cohort (*n* = 734)	No AMD cohort (*n* = 734)	Hazard ratio (95% CI)	AMD cohort (*n* = 475)	No AMD cohort (*n* = 475)	Hazard ratio (95% CI)
Ischemic heart disease	132 (18.0%)	70 (9.5%)	1.75 (1.31–2.33)	62 (13.1%)	34 (7.2%)	1.68 (1.10–2.55)
Arrhythmia-related diagnoses	320 (43.6%)	191 (26.0%)	1.69 (1.41–2.02)	70 (14.7%)	33 (6.9%)	2.04 (1.35–3.08)
Pneumonia	137 (18.7%)	79 (10.8%)	1.57 (1.19–2.07)	72 (15.2%)	41 (8.6%)	1.64 (1.12–2.41)
Stroke	85 (11.6%)	47 (6.4%)	1.66 (1.16–2.37)	42 (8.8%)	25 (5.3%)	1.49 (0.91–2.45)
Transient ischemic attack	10 (1.4%)	10 (1.4%)	1.56 (0.52–4.65)	—	—	—
Hypotension	169 (23.0%)	90 (12.3%)	1.76 (1.36–2.27)	92 (19.4%)	54 (11.4%)	1.60 (1.14–2.24)
Acute kidney injury	426 (58.0%)	308 (42.0%)	1.42 (1.23–1.64)	121 (25.5%)	69 (14.5%)	1.74 (1.30–2.34)
Dialysis initiation	214 (29.2%)	129 (17.6%)	1.59 (1.28–1.98)	108 (22.7%)	74 (15.6%)	1.36 (1.01–1.83)
Acute confusional state	51 (6.9%)	11 (1.5%)	4.19 (2.18–8.04)	—	—	—
Encephalopathy	88 (12.0%)	38 (5.2%)	2.06 (1.41–3.01)	53 (11.2%)	16 (3.4%)	3.05 (1.74–5.33)
Epilepsy	92 (12.5%)	63 (8.6%)	1.35 (0.98–1.86)	46 (9.7%)	14 (2.9%)	2.97 (1.63–5.41)
Sleep disorders	211 (28.7%)	93 (12.7%)	2.26 (1.77–2.88)	115 (24.2%)	41 (8.6%)	2.87 (2.01–4.10)
Restless legs syndrome	22 (3.0%)	10 (1.4%)	2.41 (1.07–5.42)	14 (2.9%)	12 (2.5%)	1.03 (0.48–2.24)

a Values shown as *n* (%) represent the number and proportion of patients with at least one recorded event during follow-up. Hazard ratios (HRs) were estimated using Cox proportional hazards models.

Primary cohort: propensity score–matched (*n* = 734 per group).

Subset analysis: ≥2 encounters ≥30 days apart and alive/observed at landmark (*n* = 475 per group).

Outcomes assessed from 30 days after index CKD up to 10 years. ‘—’ indicates not analyzed.

## DISCUSSION

In this large, multi-institutional cohort of young adults with stage 3–4 CKD, pre-existing AMD were associated with a higher burden of cardiovascular, renal, infectious, and neurocognitive morbidity after PSM. Associations were consistent across outcome domains and remained robust in prespecified sensitivity analyses addressing follow-up heterogeneity and surveillance bias, supporting the internal validity of the findings.

AMD were analyzed as a composite clinical exposure reflecting psychiatric disease burden documented in routine care, together with associated behavioral, psychosocial, and treatment-related factors. The study was designed to assess whether antecedent psychiatric morbidity identifies a subgroup of young CKD patients at heightened clinical risk rather than to infer outcome-specific causality, consistent with prior CKD studies linking depression and anxiety to adverse outcomes across mixed-age populations [4, 19–21].

In the primary matched analysis, all-cause mortality did not differ between groups, likely reflecting the young age of the cohort, incomplete death capture inherent to federated EHR data, and dilution of longer-term risk due to heterogeneous follow-up. To address these limitations, a prespecified subset analysis restricted to patients with documented longitudinal follow-up and delayed outcome ascertainment was performed. In this more stringently defined cohort, pre-existing AMD was associated with a higher hazard of death compared with matched controls, consistent with improved outcome capture and reduced dilution of long-term risk, and supported by the parallel accumulation of severe non-fatal outcomes in the AMD group.

The increased hazards of arrhythmia- and ischemic heart disease–related diagnoses should be interpreted in the context of real-world ICD-10 coding, which captures symptom-driven clinical encounters rather than adjudicated electrophysiological or angiographic disease. Sensitivity analyses restricted to atrial fibrillation/flutter and ventricular tachycardia yielded very low event counts, indicating that malignant arrhythmias were rare. In young adults with CKD, ischemic heart disease codes often reflect demand ischemia, autonomic instability, or microvascular dysfunction rather than premature obstructive coronary disease [22–24].

The cumulative incidence of AKI and arrhythmia-related diagnoses, while high, is biologically plausible in young adults with moderate–severe CKD followed in routine care. AKI codes capture a broad spectrum of acute renal dysfunction during intercurrent illness, and recurrent episodes are common in CKD. The parallel increases in hypotension, infection, and cardiovascular diagnoses support a phenotype of hemodynamic and autonomic vulnerability [25–28].

The higher incidence of pneumonia in patients with AMD likely reflects converging factors, including immune dysfunction, behavioral risks, medication effects (e.g. sedation or aspiration), and increased hospital exposure during AKI. Although detection bias may contribute, the magnitude of the association suggests true vulnerability.

Marked associations were also observed for neurocognitive outcomes. While acute confusional state codes represent syndrome-level diagnoses, the parallel increase in encephalopathy and established delirium precipitants, including AKI, hypotension, arrhythmia, infection, and sleep disturbance, argues against differential documentation as the sole explanation [29–31].

The higher incidence of sleep disorders and restless legs syndrome aligns with the overlap between psychiatric morbidity, sleep disturbance, and CKD-related symptoms. Sleep disruption may further exacerbate autonomic instability, blood pressure variability, and cognitive vulnerability, reinforcing bidirectional brain–kidney interactions [32, 33].

Young people with CKD face especially complex transitions [34], and the presence of mental health conditions adds further clinical burden. Effective transition programmes that support preparation, communication, and shared decision-making are associated with improved long-term outcomes [35]. Overall, our findings align with evidence from the general population showing that AMD are associated with increased risks of cardiovascular disease, infection, acute illness, and cognitive complications, even after adjustment for traditional risks [36, 37]. This study extends these observations to young adults with CKD, suggesting that psychiatric morbidity may lower the threshold for clinically adverse outcomes in this population.

## LIMITATIONS

This study has many limitations inherent to its observational design, which precludes causal inference. Exposures and outcomes were identified using routinely coded diagnoses, and misclassification is possible. Event definitions based on ICD-10 codes, particularly for arrhythmias, ischemic heart disease, and acute confusional states, capture symptom-driven clinical encounters and diagnostic evaluations rather than adjudicated disease, and therefore reflect cumulative real-world morbidity rather than precise estimates of incident pathology. Consequently, event rates may appear higher than those reported in adjudicated cohorts.

The composite definition of AMD encompasses clinically heterogeneous conditions, including milder or transient disorders and variable psychotropic treatment exposure. This heterogeneity may introduce exposure misclassification and residual confounding and is expected to bias effect estimates toward the null. Psychotropic medication use was not explicitly modeled, as medication data in federated EHR systems are time-varying and incompletely captured. Medication exposure may therefore act as a mediator or contributor to specific outcomes, warranting future analyses using time-updated exposure models.

Granular renal phenotyping was limited by incomplete availability of albuminuria, longitudinal blood pressure measurements, medication adherence data, and CKD etiology. These factors may influence outcome risk and contribute to residual confounding. CKD etiology is inconsistently coded across healthcare organizations, limiting reliable stratification without substantial misclassification. Nonetheless, etiologic heterogeneity was present in both AMD and non-AMD cohorts, and the consistency and breadth of associations across outcomes suggest a broadly relevant risk signal.

Differential detection of some outcomes, particularly delirium-related diagnoses, remains possible in patients with pre-existing psychiatric conditions. However, the parallel increase in objective physiological precipitants (including AKI, hypotension, and infection) and the concordant rise in encephalopathy, defined using neurological rather than psychiatric codes, argues against documentation bias as the sole explanation.

Follow-up duration varied across patients, and median follow-up was shorter than the maximum analytic window; therefore, estimates at later time points are less precise and long-term extrapolation should be interpreted cautiously. Follow-up-anchored sensitivity analyses were performed to reduce diagnostic-phase and surveillance bias, yielding results consistent with the primary analysis. Mortality capture in federated EHR networks may be incomplete, particularly for deaths occurring outside participating systems, likely biasing estimates toward the null; notably, a mortality difference emerged in the prespecified subset analysis with improved follow-up ascertainment.

Visit frequency and laboratory testing intervals were not standardized and reflect clinical practice across healthcare organizations. Variability in historical laboratory availability may have led to conservative misclassification of CKD onset. Clustering by healthcare organization or country could not be explicitly modeled within the TriNetX environment and may contribute to residual correlation; however, inclusion of a large number of diverse organizations reduces the likelihood of dominant site-specific bias.

Finally, this study was not designed to evaluate all potential AMD-associated conditions. Outcomes with long latency or variable ascertainment, such as cancer, were not examined. Detailed healthcare utilization metrics (e.g. visit frequency or provider type) were unavailable, limiting our ability to fully disentangle true excess morbidity from increased disease detection.

## CONCLUSION

Using global, federated real-time data, this study shows that pre-existing AMD identifies a high-risk phenotype in young adults with stage 3–4 CKD, associated with increased cardiovascular, renal, infectious, and neurocognitive morbidity and, in those with sustained follow-up, higher mortality. These findings support integrating mental health into CKD risk stratification and highlight the need for prospective studies on early, integrated interventions. Young adults transitioning to adult services represent a particularly vulnerable group, underscoring the importance of holistic transitional care models.

## Data Availability

Data supporting this study are available through TriNetX, LLC, subject to licensing and data-sharing restrictions, and are not publicly available. The analysed data are available from the corresponding author upon reasonable request.
